# Mechanism Action of Platelets and Crucial Blood Coagulation Pathways in Hemostasis

**Published:** 2017-10-01

**Authors:** Mercy Halleluyah Periayah, Ahmad Sukari Halim, Arman Zaharil Mat Saad

**Affiliations:** Reconstructive Sciences Unit, School of Medical Sciences, Universiti Sains Malaysia, 16150 Kubang Kerian, Kelantan, Malaysia

**Keywords:** Platelets, Hemostasis, Coagulation pathways, Coagulation factors, Wound healing

## Abstract

Blood is considered to be precious because it is the basic necessity for health; our body needs a steady provision of oxygen, supplied via blood, to reach billions of tissues and cells. Hematopoiesis is the process that generates blood cells of all lineages. However, platelets are the smallest blood component produced from the very large bone marrow cells called megakaryocytes and they play a fundamental role in thrombosis and hemostasis. Platelets contribute their hemostatic capacity via adhesion, activation and aggregation, which are triggered upon tissue injury, and these actions stimulate the coagulation factors and other mediators to achieve hemostasis. In addition, these coordinated series of events are the vital biological processes for wound healing phases. The aim of this review is to summarize and highlight the important pathways involved in achieving hemostasis that are ruled by platelets. In addition, this review also describes the mechanism action of platelets, including adhesion, activation, aggregation, and coagulation, as well as the factors that aid in hemostasis and wound healing.

## Introduction

 When platelets decrease in number or become malfunction, the risk of hemorrhage is very high. Platelets, which circulate within the blood, are the essential mediators that trigger the mechanical pathway of the coagulation cascade upon encountering any damage to the blood vessels. Platelets encourage primary hemostasis via three major processes: activation, adhesion and aggregation. When the integrity of the vascular endothelium is interrupted, various macromolecular elements of the vascular subendothelium become exposed and readily accessible to platelets (Table 1)^1^^-^^2^.

**Hemostasis**

Hemostasis is a process to prevent hemorrhage by arresting and keeping the blood within the damaged vessel walls. Hemostasis is a complex process that is contingent on the complex interaction of platelets, plasma coagulation cascades, fibrinolytic proteins, blood vasculatures and cytokine mediators. Upon tissue injury, the hemostatic mechanism employs a plethora of vascular and extravascular receptors, in accordance with the blood components, to seal off the impairments to the vasculature and closing it off from the encircling tissues. Normal hemostatic responses can be organized into six different important phases, which fall under three major categories of hemostasis^5^^-^^9^ (Table 2).

**Figure 1A:**
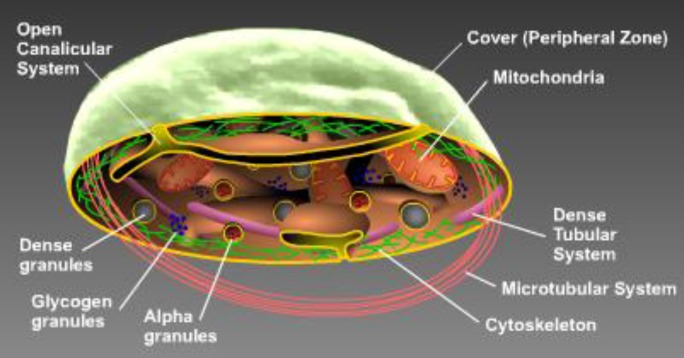
Internal structure of platelets^3^

**Table 1:** Shows the properties, structure, function and mechanism of platelets^3^^-^^4^

**Action**	**Descriptions**	
Platelets	Known as thrombocytes	
Produced from	Very large bone marrow cells called megakaryocytes	
Megakaryocytes	Develop into giant cells to release <1 X 10^3^ platelets/ megakaryocytes	
Circulate	Only 7-10 days	
Structure	2.5 µM in average normal diameter, biconvex discoid shape, sticky in nature	
Count	Normally (1.4 x 10^5^) to (4.4 x 10^5^) / µL	
Bleeding risk depending on the platelet count	≥50,000 / µL	Minimal
	20,000 – 50,000 /µL	Minor bleeding after trauma
	<20,000 / µL	Spontaneous bleeding
	<5000 / µL	Severe, possibly life threatening
**Detailed structure:** Open Canalicular Dense granules Glycogen granules Alpha granules Cytoskeleton Microtubular system Dense tubular system Mitochondria Cover	Formed by invaginations of platelets. Provide a space for platelet products to enter. Act as storage pool. Consists of non-metabolic Adenosine triphosphate (ATP) and adenosine diphosphate (ADP), serotonin and calcium. Platelets release their granules upon activation to interact with other platelet cells. Supply the energy source for platelet interactions. Act as the metabolic or cytoplasmic pool. They mainly contain Fibrinogen (Fib), thrombospodin, factor V, von Willebrand factor (vWF), beta-thromboglobuline (β-TG), and factor IV. Upon activation, platelets release their granules to interact with other platelets. The actin and myosin cytoskeleton organizes a network to sustain the platelet’s discoid shape. Upon activation, membrane receptors interlink through this network to allow platelets to change shapes into pseudopodia forms and eventually release their granule contents. Helps the actin membrane cytoskeleton maintain the discoid shape of platelets. Reorganize platelet shape changes, contract internally and granules content will release upon platelet activation. An internal smooth endoplasmic reticulum membrane, which helps to store calcium to activate platelets and aid in prostaglandin & thromboxane synthesis. Serve as an energy source because resting platelets generate their energy via oxidative phosphorylation Contains typical phospholipid bilayer membranes and glycoproteins (Gp), and membrane phospholipids that allow the coagulation proteins to interact.	
Primary function	To stop hemorrhage following vascular injury	
Other functions	Fight microbial infections, trigger inflammation to promote tumor angiogenesis and metastasis process, secrete inflammatory mediators and aid in wound therapy	
Mechanism	Under normal circumstances, platelets do not adhere to the vessel wall. However, upon tissue injury, platelets adhere to the extracellular matrix (ECM) by exchanging signals with many receptors and mediators to coordinate rolling of platelets to adhere at the sites of vascular injury. Firm platelet adhesion stimulates a signaling mechanism mediates via tyrosine kinases and G-protein coupled receptors, which supports platelet activation, resulting in granule release and increasing the number of platelets. Platelet adherence and activation initiate platelet aggregation to provide a procoagulant surface engaged in the formation of a fibrin-rich hemostatic plug at the injured area. Activated platelets stimulate endothelial cells to synthesize and secrete molecules that control and limit the formation of a thrombus.	
Stained smear	Appears as a dark purple spot on Geimsa-stained peripheral blood smear. Used to study the size, shape, qualitative number and clumping. Upon biomaterial adherences, platelets can be fixed in 2.5% glutaraldehyde for viewing under a scanning electron microscope (SEM)	
Shape changes		

**Figure 1:**
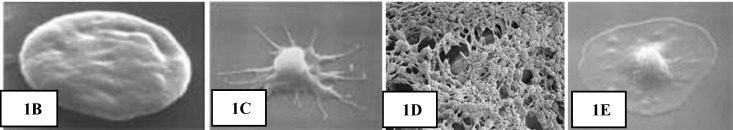
[**(B) **Platelet in resting mode **(C)** Activated platelets change into a pseudopodia shape **(D)** Aggregated platelets **(E)** Platelet spreading]

**Table 2:** Represents the mechanical pathway of three different types of hemostasis

**Type of hemostasis**	**Mechanism**
Primary hemostasis	Blood vessel contraction /vasoconstriction Platelet plug formation upon platelet adhesion and aggregation
Secondary hemostasis	Activation of the coagulation cascade Deposition and stabilization of fibrin
Tertiary hemostasis	Dissolution of fibrin clot Dependent on plasminogen activation

**Vasoconstriction**

**Figure 2.**
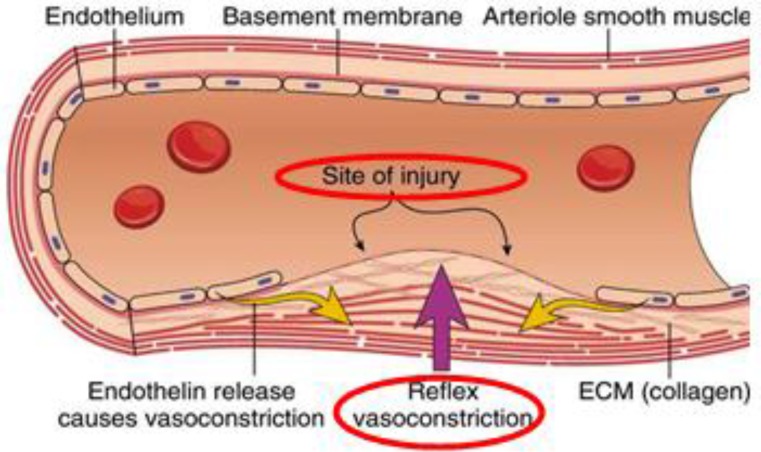
Vasoconstriction phase. Primary hemostasis is characterized by vasoconstriction, which is the initial phase for stopping the blood flow^10^.

Vascular spasm occurs whenever there is an injury or damage to the blood vessels. This will trigger a vasoconstriction, which could eventually stop the blood flow. This reaction can be responded within 30 minutes, and is localized to the injured area. At this stage, exposed collagen fibers will release ATP and other inflammatory mediators to recruit macrophages. In addition, the ECM becomes highly thrombogenicity, promoting platelet adhesion and aggregation^10^^-^^12^.

**Platelet plug formation**

**Figure 3:**
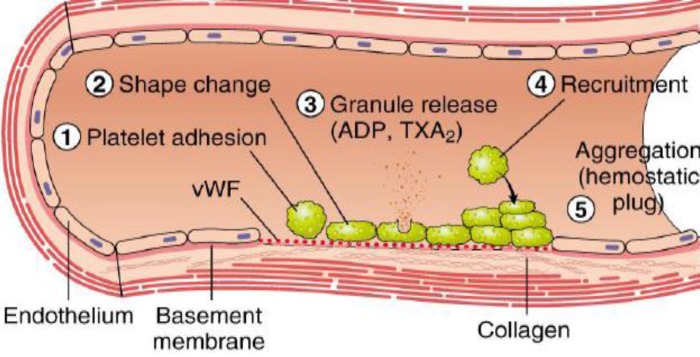
Platelet plug formation. Injuries on the endothelial cells highly exposes to thrombogenic, subendothelial ECM to ease platelet adherences and activation. Platelet activation triggers platelet shape changes by releasing secretory granules. Released secretary granules will recruit additional platelets to form the platelet plug, which is referred to as primary hemostasis^10^.

Following vasoconstriction, exposed collagen from the damaged surface will encourage platelets to adhere, activate and aggregate to form a platelet plug, sealing off the injured area.

**Platelet adhesion**

The platelet adhesion mechanism is generally supported by the particular interactions between the membrane receptors and absorbed plasma proteins. The platelet membrane receptors are enriched with Gp receptors embedded in the phospholipid bilayer, including tyrosine kinase receptors, integrins, leucine rich receptors; G- protein coupled transmembrane receptors, selectins and immunoglobulin domain receptors. These are the important proteins involved to facilitate hemostatic function by mediating the interactions within cell-platelet and platelet-substrates^13^^-^^15^. The initial event that occurs upon hemostasis is the rolling and adherence of the platelets to the exposed subendothelium. Platelet adhesion is mediated by von Willebrand Factor (vWF) that binds to Gp Ib-IX in the platelet membrane. vWF is a blood Gp that serves as an adhesive protein, which could bind to other proteins, especially factor VIII at the wound sites ^10^^, ^^16^^-^^17^^,^^19^.

**Platelet activation**

**Figure 4:**
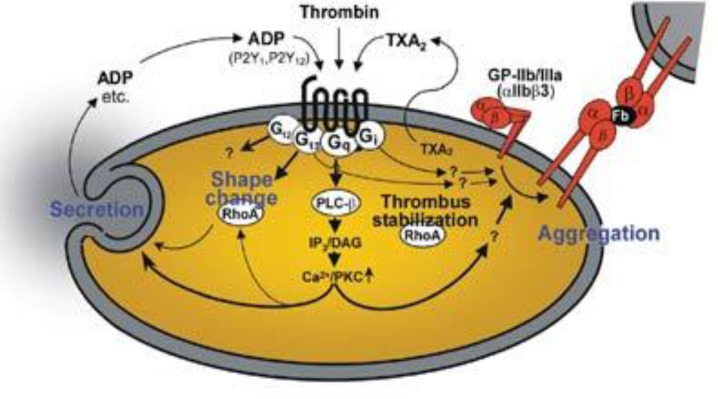
Platelet activation process. The schematic diagram portrays the internal organelles with prominent crucial storage contents that are involved in platelet activations and aid in platelet aggregation^20^.

A variety of stimuli can activate platelets. Platelet cells can also be activated upon biomaterial surface stimulation. Adhered platelets undergo degranulation and release cytoplasmic granules that contain serotonin, platelet activating factors and ADP. ADP is an important physiological agonist stored in the dense bodies of platelets that play an essential function in normal hemostasis and thrombosis. Platelets are activated to change shapes into a pseudopodal form upon the adhesion to the injured area which will activate the collagen receptors on their surface membrane, named GpIIbIIIa, to undergo release reactions. The GpIIbIIIa complex, organized through calcium-dependent association of GpIIb and GpIIIa that is a necessary step in platelet aggregation and endothelial adherence^21^^-^^23^. At the same time, platelets tend to synthesize and discharge thromboxane A2 (TXA2), aiding in vasoconstriction and platelet aggregation. In addition, GpIIbIIIa integrins and P-selectin move from the α-granule membrane to the platelet membrane to support platelet aggregation. Additionally, these are the receptors that could act as the catalytic surface and facilitate the hemostasis process^21^^-^^24^.

**Platelet aggregation**

**Figure 5:**
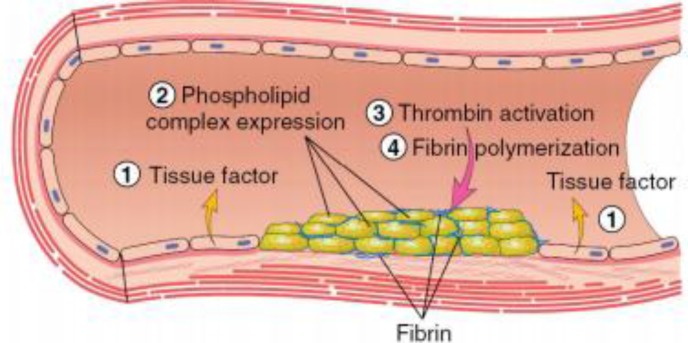
Platelet aggregation phase. Tissue factor (TF) also known as factor III and thromboplastin, is a membrane-bound procoagulant. TF acts with factor VII as the major in vivo initiator of the coagulation cascade to generate thrombin. Thrombin adheres with circulating Fib and convert into insoluble fibrin by forming a fibrin network. This fibrin network strengthens the initial platelet plug^10^.

Platelet aggregation begins once platelets become activated, triggering the GpIIbIIIa receptor (50-100/platelets), which attach to vWF or Fib. Each activated platelet extends pseudopods, clumping and becoming aggregated. These activations are further heightened by the generation of thrombin via the hemostasis mechanism. Platelet aggregation promotes a primary platelet plug. The ADP receptor interconnects with a family of ADP receptors (P2Y1 and P2Y12), which could be detected on platelets as helping with aggregation. P2Y1 receptors assist in stimulating the initial platelet shape changes and platelet aggregation. At the same time, P2Y12 is an important mediator for blood clotting. It increases significantly, responding to ADP to complete the aggregation process. Eventually, the formed platelet plug ought to be stabilized by the formation of fibrin^10^^, ^^25^^-^^28^.

**The coagulation mechanism**

**Figure 6:**
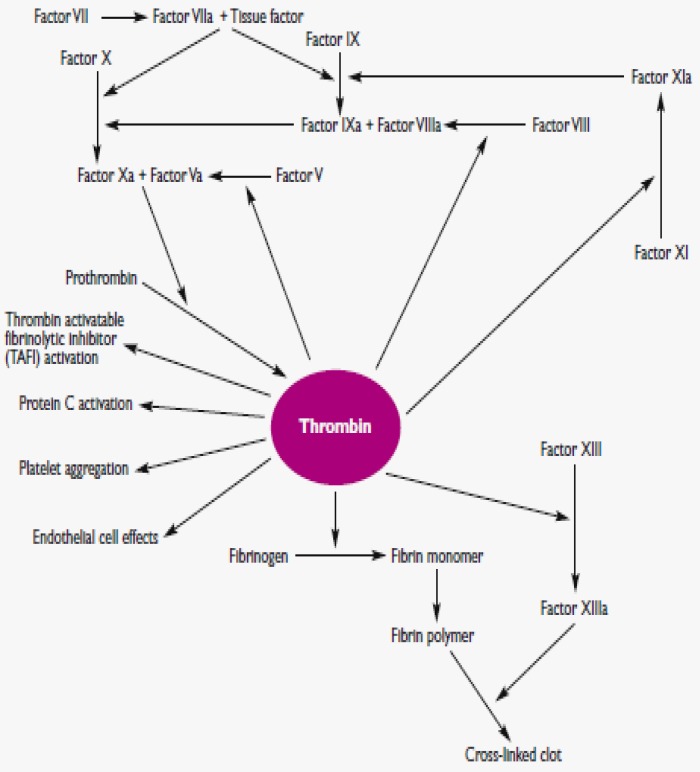
Coagulation mechanism; Thrombin plays a vital role in generating cross-linked fibrin by cleaving Fib to fibrin and activating several other coagulation factors. Thrombin also modulates other important cellular activities via protease-activated receptors. Simultaneously, it will directly increase the platelet aggregation and the production of TXA2 to express adhesion molecules^29^.

Approximately fifty significant substances affect the blood coagulation mechanisms. The blood coagulation cascade of secondary hemostasis mainly consists of two main pathways: (i) intrinsic (contact activation pathway) (ii) extrinsic (TF pathway). The blood clotting process can be classified into three important steady steps as follows: (i) involvement of a complex cascade, triggering the chemical reactions that are mediated by the coagulation factors that respond to form fibrin strands for consolidating the platelet plugs; (ii) the conversion of prothrombin (PT) into thrombin, which is catalyzed by the PT activator; and (iii) conversion of Fib into fibrin, which eventually enmeshes the plasma, platelets and blood cells to build a firmer clot (Figure 6; Table 3)^29^^-^^31^. 

**Extrinsic pathway**

The newer blood coagulation cascade model was well elaborated in a Jerry B. Lefkowitz. Thrombin was portrayed as the center of the coagulation universe. All the coagulation factors involved in the hemostasis process feed into the regulation and control of thrombin generation, which then forms clots at the sites of vascular injury. Thrombin is a proteolytic enzyme derived from PT, which aids in the process of forming blood clots by catalyzing the conversion of Fib to fibrin. The modified intrinsic coagulation cascade, which displayed in Figure 6, is different from the older one and lacks the significance of factor XII and prekallikren. Apparently, these proteins are not considered to play a crucial role in the coagulation process in vivo. There are two major processes that could initiate the blood clotting mechanism. They are extrinsic and intrinsic pathways. 

First, TF binds to factor VII or activated FVIII (FVIIIa) in 1:1 ratio complex. A limited proteolysis process extends to the TF / FVIIIa complex, which activates factor X or factor IX, further activating factor X/ FIX and activating serine proteases via cleaving an activation peptide. Proteolysis is the hydrolysis process that involves the breakdown of proteins into smaller polypeptides. Once the extrinsic pathway is triggered, the activation of factor X/ IX in the TF/ FVIIa complex is instantly inhibited by the TF pathway inhibitor (TFPI), which is generated from endothelial cells. Freshly activated factor IXa subsequently adheres to its cofactor, factor VIIIa, upon the phospholipid surface to stimulate the tenase complex which results in the activation of factor X to factor Xa. 

Finally, the common pathway for thrombin activation is initiated via the activation of factor Xa. The activated factor Xa merges with the cofactor, activated factor V (FVa) and calcium on the phospholipid surfaces to construct the prothrombinase complex. This complex eventually helps to convert PT to thrombin by cleaving the PT, which is the activation peptide. Thrombin activation will be generated to a very minor extent by the extrinsic pathway, which is adequate and crucial to initiate the coagulation cascade which subsequently triggers and expands thrombin generation via the intrinsic pathway^29^^-^^30^^, ^^32^.

**Intrinsic pathway**

The activation of factor XI to factor XIa, and more thrombin generated via factor IXa and factor VIIIa leads to the activation of factor X, which is involved in the intrinsic pathway. Factors V and VIII, which are partially proteolyzed or activated, are known to be involved in and facilitate the hemostasis process. Subsequently, the activation of factors V and VIII by thrombin triggers more mechanical action of the coagulation pathway by enhancing the bioactivity of tenase and prothrombinase complexes. 

As described in Table 3, factor I (Fib) plays a crucial role in forming a fibrin clot to seal the injured area with fibrin meshes. Fib typically consists of 3 globular domains, which is the central E domain attached or flanked by two identical D domains. At this stage, thrombin sticks to fibrinopeptides A and B, which are derived from the Aα and Bβ chains, to build a fibrin monomer. These monomers gather into protofibrils in a half-distributed manner, which is stabilized by the non-covalent interactions among fibrin molecules. Eventually, the protofibrils are obliquely organized into dense fibrin networks to form a temporary fibrin clot that is not covalently crosslinked. 

Nevertheless, to form a stable blood clot, thrombin needs to activate factor XIII to the transglutaminase enzyme activated factor XIIIa. Factor XIIIa will stimulate the glutamic acid and lysine side chains, producing a stable clot. Factor XIIIa is the fibrin stabilizing factor of the blood coagulation system that crosslinks with fibrin. Furthermore, factor XIIIa also plays a significant role towards tissue repair and the angiogenesis process (Figure 6)^29^^-^^30^^,^^32^^,^^35^.

**Tertiary hemostasis**

Once the fibrin clot has been formed, the activated platelets will be well organized and take position to contract their intracellular actin or myosin cytoskeleton. The intracellular actin network will directly connect to the integrin GpIIbIIIa and Fib receptor internally. Subsequently, the external component of GpIIbIIIa will adhere to the fibrin network of the blood clot, making the clot compact and decreasing the clot volume slowly, which is called clot retraction. A plasminogen activator is a serine protease that converts plasminogen to plasmin to promote fibrinolysis by cutting and degrading the fibrin networks. Plasmin slashes off the fibrin meshes formed around the wounded area, resulting in the formation of other circulating fragments that are cleared by other proteases or by the kidney and liver. The clot resolution mechanism helps in clearing the injured and obstructed vessels, regenerating blood flow that is directed to the normal blood flow pathway. GpIIbIIIa disrupts the fibrin binding capacity with platelets and complete the clot resolution process^29^^-^^30^^,^^36^^-^^37^.

**Wound healing**

**Figure 7:**
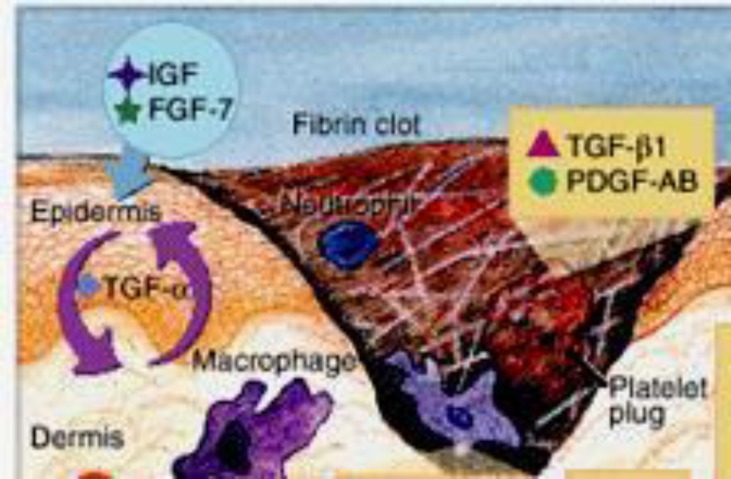
Wound healing phase. This figure shows one of the important modified paradigm of the wound healing phase, that involves two important mediators (TGF-β1 and PDGF-AB) upon the formation of a platelet plug to seal the wound with the aid of a fibrin clot. These signaling molecules are released to initiate the proliferative phase of the wound healing process^39^^-^^40^.

Wound healing is an innate revitalizing response in tissue injuries, and the interaction of the cellular mechanical pathway events results in resurfacing, reconstitution and refurbishment of cells on the injured surface area. The healing process can be explained in three different phases followed by hemostasis, including inflammation, proliferation and maturation^40^. While platelet play its crucial role in clot formation as part of the hemostasis process, the inflammatory cells aid in the inflammatory phase, which is followed by the proliferative phase, consisting of epithelialization, fibroplasia and angiogenesis. In addition, wound healing is also regulated by cytokines and growth factors. Collagen tends to form tight cross-links with other collagen 

**Table 3:** illustrates the engagement and detailed explanation of coagulation factors, that aid in the blood coagulation cascade

**Factor**	**Name**	**Source**	**Pathway**	**Description**	**Function**
I	Fib	Liver	Common	Plasma glycoprotein; Molecular Weight (MW)= 340 kilodaltons (kDa)	Adhesive protein which aids in fibrin clot formation.
II	Prothrombin	Liver	Common	Vitamin K-dependent serine protease; MW= 72 kDa	Presence in the activated form and the main enzyme of coagulation
III	Tissue factor	Secrete by the damaged cells and platelets	Extrinsic and Intrinsic	Known as thromboplastin; MW= 37 kDa	Lipoprotein initiator of the extrinsic pathway
IV	Calcium ions	Bone and gut	Entire process	Required for coagulation factors to bind to phospholipid (formerly known as factor IV)	Metal cation which is important in coagulation mechanisms
V	Proaccererin / Labile factor	Liver and platelets	Intrinsic and extrinsic	MW = 330 kDa	Cofactor for the activation of prothrombin to thrombin (prothrombinase complex)
VII	Proconvertin (stable factor)	Liver	Extrinsic	MW = 50 kDa; vitamin K- dependent serine protease	With tissue factor, initiates extrinsic pathway (Factor IX and X)
VIII	Antihemophilic factor A (cofactor)	Platelets and endothelium	Intrinsic	MW = 330 kDa	Cofactor for intrinsic activation of factor X (which it forms tenase complex)
IX	Christmas factor / Antihemophilic factor B (plasma thromboplastin component)	Liver	Intrinsic	MW = 50 kDa; vitamin K- dependent serine protease	Activated form is enzyme for intrinsic activation of factor X (forms tenase complex with factorVIII)
X	Stuart-Prower factor (enzyme)	Liver	Intrinsic and extrinsic	MW = 58.9 kDa; vitamin K- dependent serine protease	Activated form is the enzyme for final the common pathway activation of prothrombin (forms prothrombinase complex with factor V)
XI	Plasma thromboplastin antecedent	Liver	Intrinsic	MW = 160 kDa; serine protease	Activates intrinsic activator of factor IX
XII	Hageman factor	Liver	Intrinsic; (activates plasmin)	MW = 80 kDa; serine protease	Initiates activated partial thromboplastin time (aPTT) based intrinsic pathway; Activates factor XI, VII and prekallikrein
XIII	Fibrin stabilizing factor	Liver	Retards fibrinolysis	MW = 320 kDa; Crosslinks fibrin	Transamidase which cross-links fibrin clot

and protein molecules in the maturation phase as well as completes the wound healing mechanism^40^^-^^41^.

Cytokine is a protein mediator that is released from various cells. They can adhere to the cell surface receptors to trigger cell reactions and play a significant role in the wound healing phase. There are many cytokines responsible for wound healing, but Transforming growth factor-beta 1 (TGF-β1) and platelet derived growth factor- AB (PDGF-AB) are the main cytokines acting on cell proliferation and maturation. TGF-β1 helps to stimulate fibroblast proliferation and the production of proteoglycans, collagen and fibrin. It is also noted that TGF-β1 assists in decreasing scar formation and preventing inhibition of healing by glucocorticoids. On top of that, TGF-β1 supports the expression of specific cytokines in T cells to promote cell proliferation, especially while the cells still undergo the immature phase^42^^-^^44^. 

Meanwhile, PDGF-AB plays a specific role in blood vessel formation, which is addressed as an angiogenesis process. The angiogenesis process involves the growth of blood vessels from the existing blood vessel tissue. This is followed by the proliferation phase. The subsequent maturation phase replaces and remodels the structure of collagen type 3 to type 1. This maturation phase could last for a year or more than a year, depending on the severity of the wound type (Figure 7)^40^^, ^^43^^-^^45^^,^^47^. 

## CONCLUSION

 Platelets are the smallest blood component, that capable to act as a fundamental role in thrombosis and hemostasis. Initial platelet adhesion, activation and aggregation upon tissue injury, stimulates coagulation factors and other mediators to achieve hemostasis. In addition, these coordinated events are the vital biological processes towards wound healing upon the tissue damage. The history on the investigation of platelet function began 100 years ago, and has recently become increasingly important for monitoring hemorrhage, antiplatelet activity, platelet dysfunctions and coagulation factors. Understanding the platelet thrombogenicity cascade is very beneficial to reducing the hematological complications. As platelets are remarkable cells that arrest bleeding and circulate within the human body, the search for artificial platelet substitutes should be part of the next attempt or search to decrease platelet-related morbidities and mortalities. 
